# Improved haemodynamic stability and cerebral tissue oxygenation after induction of anaesthesia with sufentanil compared to remifentanil: a randomised controlled trial

**DOI:** 10.1186/s12871-020-01174-9

**Published:** 2020-10-07

**Authors:** Marieke Poterman, Alain F. Kalmar, Pieter L. Buisman, Michel M. R. F. Struys, Thomas W. L. Scheeren

**Affiliations:** 1 Department of Anaesthesiology, University Medical Center Groningen, Hanzeplein 1, PO Box 30 001, 9700 RB Groningen, The Netherlands; 2 Department of Anaesthesiology, AZ Maria Middelares Gent Buitenring Sint-Denijs 30, 9000 Ghent, Belgium

**Keywords:** Induction of anaesthesia, Sufentanil, Remifentanil, Atropine, Haemodynamics, Tissue oxygenation

## Abstract

**Background:**

Balanced anaesthesia with propofol and remifentanil, compared to sufentanil, often decreases mean arterial pressure (MAP), heart rate (HR) and cardiac index (CI), raising concerns on tissue-oxygenation. This distinct haemodynamic suppression might be attenuated by atropine. This double blinded RCT, investigates if induction with propofol-sufentanil results in higher CI and tissue-oxygenation than with propofol-remifentanil and if atropine has more pronounced beneficial effects on CI and tissue-oxygenation in a remifentanil-based anaesthesia.

**Methods:**

In seventy patients scheduled for coronary bypass grafting (CABG), anaesthesia was induced and maintained with propofol target controlled infusion (TCI) with a target effect-site concentration (Cet) of 2.0 μg ml^− 1^ and either sufentanil (TCI Cet 0.48 ng ml^− 1^) or remifentanil (TCI Cet 8 ng ml^− 1^). If HR dropped below 60 bpm, methylatropine (1 mg) was administered intravenously. Relative changes (∆) in MAP, HR, stroke volume (SV), CI and cerebral (SctO_2_) and peripheral (SptO_2_) tissue-oxygenation during induction of anaesthesia and after atropine administration were analysed.

**Results:**

The sufentanil group compared to the remifentanil group showed significantly less decrease in MAP (∆ = − 23 ± 13 vs. -36 ± 13 mmHg), HR (∆ = − 5 ± 7 vs. -10 ± 10 bpm), SV (∆ = − 23 ± 18 vs. -35 ± 19 ml) and CI (∆ = − 0.8 (− 1.5 to − 0.5) vs. -1.5 (− 2.0 to − 1.1) l min^− 1^ m^− 2^), while SctO_2_ (∆ = 9 ± 5 vs. 6 ± 4%) showed more increase with no difference in ∆SptO_2_ (∆ = 8 ± 7 vs. 8 ± 8%). Atropine caused higher ∆HR (13 (9 to 19) vs. 10 ± 6 bpm) and ∆CI (0.4 ± 0.4 vs. 0.2 ± 0.3 l min^− 1^ m^− 2^) in sufentanil vs. remifentanil-based anaesthesia, with no difference in ∆MAP, ∆SV and ∆SctO_2_ and ∆SptO_2_.

**Conclusion:**

Induction of anaesthesia with propofol and sufentanil results in improved haemodynamic stability and higher SctO_2_ compared to propofol and remifentanil in patients having CABG. Administration of atropine might be useful to counteract or prevent the haemodynamic suppression associated with these opioids.

**Trial registration:**

Clinicaltrials.gov on June 7, 2013 (trial ID: NCT01871935).

## Background

Balanced general anaesthesia with a combination of propofol and remifentanil, as compared to propofol with other opioids like sufentanil, provides some beneficial pharmacological properties, such as a fast and reliable induction and reversal of anaesthesia, swift postoperative recovery and avoidance of postoperative nausea and vomiting [1, 2]. However, vasodilation and cardiac depression caused by this type of anaesthesia often induces a decrease in mean arterial pressure (MAP), heart rate (HR) and cardiac index (CI), raising concerns on maintaining an adequate tissue oxygenation [3–5].

Haemodynamic suppression is often observed after induction of general anaesthesia with any combination of hypnotics and opioids. Because of the distinct pharmacodynamic differences of remifentanil compared to other opioids such as sufentanil or fentanyl, different haemodynamic side effects may occur with differential effects on tissue oxygenation [6–8]. In addition to a strong suppressive effect on the heart rate, remifentanil dose-dependently depresses the sinus and AV node function, and significantly prolongates the sinus node recovery time, sino-atrial conduction time and Wenckebach cycle length, resulting in an inhibition of both the intra-atrial conduction and sinus node automaticity [7, 8]. This distinct effect of remifentanil on cardiac conduction might have a particularly significant negative impact on the cardiac index compared to sufentanil.

Perioperative maintenance of adequate tissue oxygenation has been associated with less postoperative complications, such as reduction in surgical wound infections and length of hospital stay, and is specifically important for high-risk surgical patients, including patients scheduled for coronary artery bypass grafting surgery [9–11].

Beneficial effects of atropine, not only on MAP and HR, but also on CI during propofol-remifentanil anaesthesia in patients undergoing non-cardiac surgery have been reported [12]. However, this may not be equally valid during anaesthesia with propofol combined with other opioids, such as sufentanil, and in patients undergoing cardiac surgery. Positive inotropic agents such as dopamine, dobutamine or ephedrine are in general preferably used in cardiac surgery. However, the prominent effect of remifentanil on the cardiac conduction suggests that it has a direct parasympathicomimetic effect, thereby making a beneficial effect of atropine more likely. In these circumstances, atropine may not only mitigate bradycardia and increase the arterial blood pressure, but also increase CI and tissue oxygenation.

We therefore hypothesised that induction of anaesthesia with propofol and sufentanil results in different haemodynamic suppression and tissue oxygenation values compared to anaesthesia with propofol and remifentanil in calculated equipotent dosages. Also, we expected that atropine will have different effects on the haemodynamic suppression and tissue oxygenation in both groups.

## Methods

**Fig. 1:**
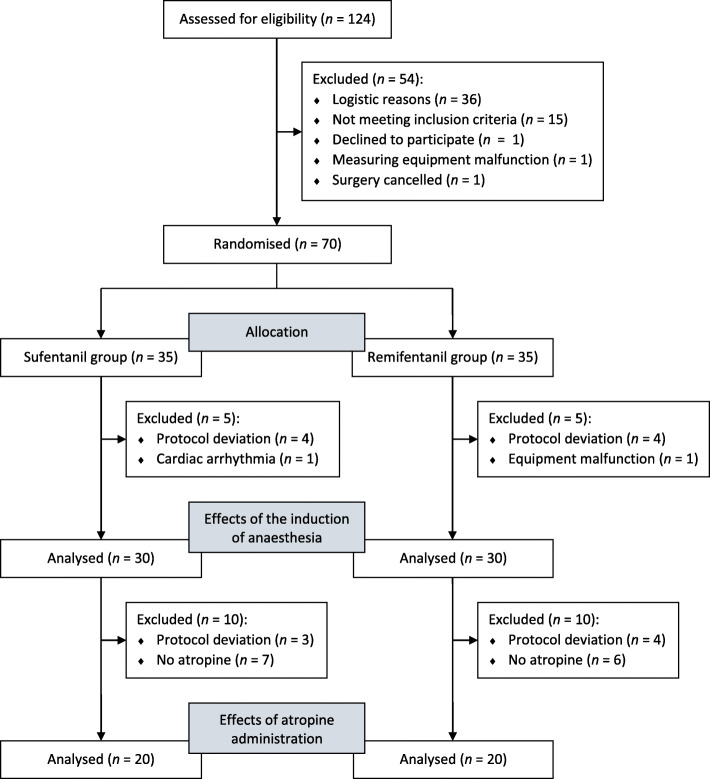
CONSORT flow diagram

Ethical approval for this study was provided by the Ethical Committee of University Medical Center Groningen, Groningen, The Netherlands. After registration at ClinicalTrials.gov (Ref: NCT01871935), all patients ≥18 years scheduled for elective off-pump performed Coronary Artery Bypass Grafting (CABG) surgery between 17 June 2013 and 1 October 2013 were assessed for eligibility for this interventional, prospective randomised controlled trial according to the CONSORT group statement (Fig. 1) [13]. Patients undergoing emergency surgery or with a contraindication for atropine administration, such as severe aortic valve stenosis, were excluded. In addition, morbidly obese patients (body mass index > 35 kg m^− 2^) were excluded, since anaesthesia with target controlled infusion (described below) in this patient category is not reliable [14]. There was no selection made based on age, gender, co-morbidity or ethnic background. Following written informed consent, all included patients were randomly assigned to the sufentanil or remifentanil group using the sealed opaque envelope technique. Randomisation was unblinded only after finishing the full data collection. The complete study adhered to CONSORT guidelines [13].

### Study protocol

Based on theoretical drug interaction pharmacodynamic models [15], for propofol/sufentanil and propofol/remifentanil, equipotent opioid targeted effect site concentrations (Cet) were calculated to be 0.48 ng ml^− 1^ (Gepts model) and 8 ng ml^− 1^ (Minto model) respectively.

Before induction of anaesthesia, adequate pre-oxygenation of the patient’s lungs was performed via a face mask. Anaesthesia was induced as follows: according to randomisation, a syringe pump with sufentanil (target-controlled infusion (TCI), targeted effect site concentration (Cet) 0.48 ng ml^− 1^, Gepts model) or dose of remifentanil (TCI Cet 8 ng ml^− 1^, Minto model) was started, followed in both cases by a syringe pump containing propofol (TCI Cet 2.0 μg ml^− 1^, Schnider model) [16–18].

Doses were left unchanged throughout the study period. Maximal reproducibility of the anaesthetic pharmacological condition was pursued by using these propofol and opioids effect site concentrations to obtain a tolerance of laryngoscopy in 95% of patients as predicted by a hierarchical interaction model [3, 19, 20]. After loss of consciousness and achievement of a bispectral index value between 40 and 60, rocuronium (0.6 mg kg^− 1^) was administered and the patient’s trachea was intubated. Mechanical ventilation was started in the volume control mode (tidal volume: 8 ml kg^− 1^) with an O_2_/air mixture (F_i_O_2_ 0.4) and a positive end-expiratory pressure of 5 cmH_2_O. The respiratory rate was adjusted to keep end-tidal carbon dioxide partial pressure between 4.5 kPa (34 mmHg) and 5.5 kPa (42 mmHg).

If the HR dropped below 60 beats per minute (bpm), methylatropine (1 mg) was administered intravenously. In those cases where the patient had a baseline HR < 60 bpm prior to the induction of anaesthesia, methylatropine (1 mg) was administered when HR dropped more than 10% below the awake HR value.

If the MAP dropped below 80% of the baseline value, without the above-mentioned indications for administration of atropine or from 3 min after the administration of atropine, other appropriate measures (e.g. fluid or vasopressor administration) were taken.

### Haemodynamic monitoring

Upon arrival in the operating theatre standard monitoring equipment was connected to the patient: ECG, pulse oximetry and non-invasive blood pressure monitoring (Philips IntelliVue MX800, Philips, Eindhoven, The Netherlands) and routine physiological measurements and monitoring was started. Subsequently two large peripheral intravenous cannulas and an invasive arterial catheter were inserted for invasive blood pressure monitoring, as is usual practice in cardiothoracic anaesthesia.

In addition, a FloTrac sensor (Edwards Lifesciences, Irvine, United States) was connected to the arterial line and subsequently to the Vigileo monitor (Edwards Lifesciences). The FloTrac-Vigileo system analysis the arterial pressure waveform for calculation of the stroke volume (SV) and CI [21]. Two INVOS™ Cerebral Oximeter (Medtronic, Minneapolis, United States) sensors were placed on the patients’ forehead to record cerebral tissue oxygenation (SctO_2_) [22] and an InSpectra™ (Hutchinson Technology Inc., Hutchinson, United States) probe was positioned on the thenar eminence to measure the peripheral tissue oxygenation (SptO_2_) [23]. Both devices rely on near-infrared spectroscopy technology [24]. Briefly, the sensors positioned on the patient’s skin emit light in several device-specific wavelengths from the near infrared spectrum. Based on the ratio of oxygenated and deoxygenated haemoglobin in the underlying tissue, these light signals are partially absorbed and partially reflected to the sensors [25]. Changes relative to baseline tissue oxygenation are significantly correlated with oxygen delivery and perfusion deficits [26].

### Data registration and analysis

All data from routine physiological measurements and from the additional study devices were recorded continuously (sampling rate of 1 Hz) on the central hospital server using a cardiothoracic specific data management system (Carola, University Medical Center Groningen, Groningen, The Netherlands).

The electronic data were imported into Microsoft Excel 2016® (Microsoft, Redmond, United States) for synchronization and analysis. After graphical representation, absent values caused by artifacts were corrected by interpolation during a visual inspection of the data plots. Subsequently, we calculated the rate pressure product (RPP) by multiplying the systolic arterial pressure and heart rate. RPP is a surrogate measure of myocardial oxygen uptake [27, 28].

Additionally, a 30 s running median with 15 s steps was calculated for all studied variables. The evolution of the absolute values and of the changes relative to baseline was plotted from 1 min before the induction of anaesthesia until 6 min afterwards, and from 1 min before the administration of atropine until 4 min afterwards. This covers the time to achieve relative steady state for all study variables.

### Statistical analysis

The primary outcome measure was the change in CI around the moment of atropine administration. Secondary outcome measures were the changes in MAP, SctO_2_ and SptO_2_.The sample size calculation was based on the primary endpoint, CI. A mean difference in CI of 10% between sufentanil and remifentanil was considered clinically relevant. We expected the standard deviation to be 10% in both groups. A type I error probability of 0.05 and a power of 0.95 delivers a total sample size of 54 [29]. A supplemental 25% of patients were included in each group to anticipate invalid data recordings and patients not meeting the criteria for atropine administration, making a total of 70 patients (35 patients in each group).

Statistical analysis was performed in SPSS version 23 (IBM Corporation, Armonk, United States). Categorical variables are given as number of patients and analysed with the Chi-square test or the Fisher’s exact test. Continuous data are expressed as mean ± SD or median (IQR), depending on the Kolmogorov-Smirnov tested normality. Differences between groups were tested on absolute values during the induction of anaesthesia and during atropine administration, Time = 0 min (T_0_), and at the moment of steady state (after the time to peak of propofol, sufentanil and remifentanil or atropine [30, 31], Time = 6 min (T_6_) for the induction of anaesthesia and Time = 4 min (T_4_) for the administration of atropine, and on relative changes (∆ = value (T_6_ or T_4_) – value (T_0_)). To compare continuous variables of the different groups, the unpaired student *t*-test was used for parametric variables, and the Mann-Whitney *U* test for non-parametric variables. Comparison of the values of the haemodynamic variables from the same group between T_0_ and T_6_ (for the induction of anaesthesia) or T_4_ (for the administration of atropine) was performed using a paired *t*-test and Wilcoxon signed rank test for parametric and non-parametric variables, respectively. Two-tailed tests were performed and statistical significance was defined as *P* < 0.05 (after Bonferroni correction for multiple comparisons) in all cases.

## Results

A total of 70 patients were included in this double blind, randomised controlled trial and subsequently allocated to the sufentanil or remifentanil group (Fig. 1). From these, we had to exclude 10 patients before the analysis of the haemodynamic effects of the induction of anaesthesia due to deviation from the study protocol (*n* = 8), cardiac arrhythmia (*n* = 1) or measuring equipment malfunction (*n* = 1). An additional 20 patients were excluded from the analysis of the effects of atropine administration by reason of study protocol deviation (*n* = 7) and not meeting the criteria for atropine administration (*n* = 13). Protocol deviations comprises usage of a different dose of hypnotics and/or opioids than described in the protocol, additions to anaesthesia (e.g. volatile anaesthesia) or the use of additional haemodynamic support (fluid or vasopressor administration) during the measurement periods (from 1 min before induction of anaesthesia or administration of atropine until respectively 6 or 4 min thereafter). Table 1 shows the baseline characteristics of the patients per analysis in both groups. American Society of Anaesthesiologists classification of Physical Health was not included in this table, because all patients belong to American Society of Anaesthesiologists class III. There were no significant between-group differences for both analyses.

**Table 1:** Baseline characteristics of the patients per analysis in the sufentanil and remifentanil group

	Induction of anaesthesia		Atropine administration	
	Sufentanil (*n* = 30)	Remifentanil (*n* = 30)	Sufentanil (*n* = 20)	Remifentanil (*n* = 20)
Age (years)	64 ± 8	67 ± 8	66 ± 9	70 ± 7
Sex, male	23	23	18	16
Height (cm)	174 ± 10	174 ± 11	175 ± 8	175 ± 11
Weight (kg)	81 ± 16	78 ± 11	82 ± 14	83 ± 11
BMI (kg m^− 2^)	27 ± 4	26 ± 3	27 ± 4	27 ± 3
Medical history				
Arterial hypertension	20	21	13	15
Peripheral vascular disease	4	2	4	0
Diabetes mellitus	11	7	8	5
Chronic obstructive pulmonary disease	8	8	6	4
Relevant medication				
Beta blocking agents	27	29	18	18
Angiotensin receptor blockers	7	4	6	1
Angiotensin-converting enzyme inhibitors	14	19	9	13

Continuous variables are reported as mean ± SD and categorical data as numbers

### Haemodynamic effects after the induction of anaesthesia

**Fig. 2:**
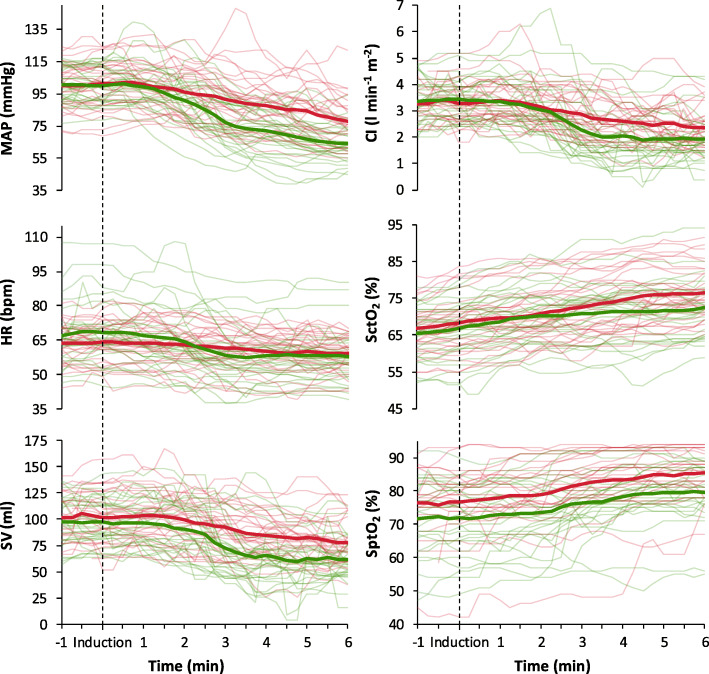
Mean values (thick lines) and individual patient data (thin lines) of the investigated haemodynamic variables: mean arterial pressure (MAP), heart rate (HR), stroke volume (SV), cardiac index (CI), cerebral tissue oxygen saturation (SctO_2_) and peripheral tissue oxygen saturation (SptO_2_). Red lines represent the sufentanil group and green lines the remifentanil group. Graphs are shown from 1 min before until 6 min after the induction of anaesthesia

**Fig. 3:**
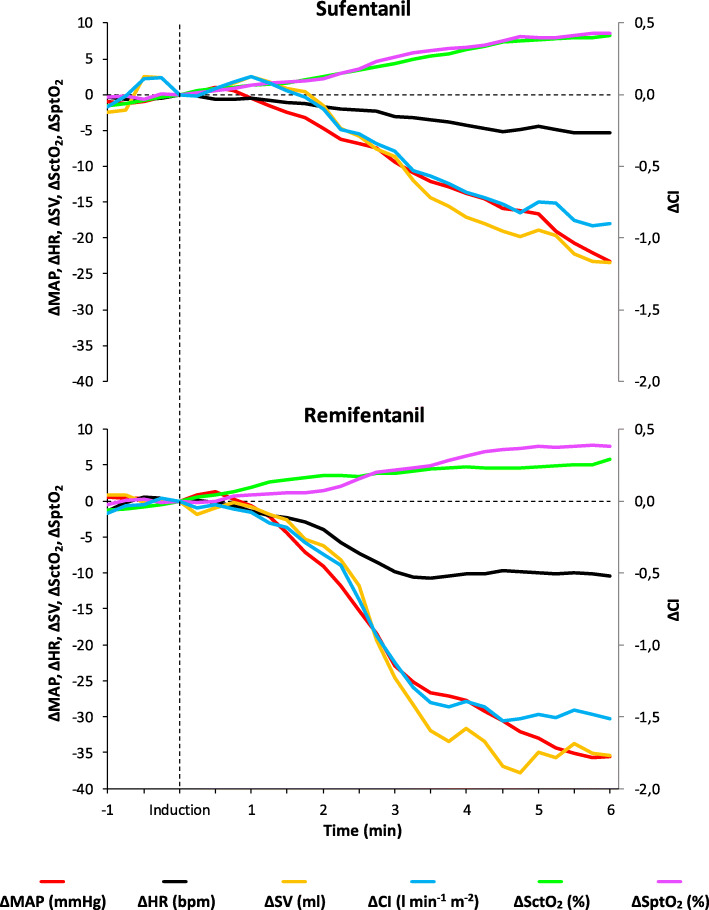
Mean relative changes of the investigated haemodynamic variables during the induction of anaesthesia (from 1 min before until 6 min after). Mean arterial pressure (ΔMAP), heart rate (ΔHR), stroke volume (ΔSV), cardiac index (ΔCI), cerebral tissue oxygen saturation (ΔSctO_2_) and peripheral tissue oxygen saturation (ΔSptO_2_)

The course over time of the investigated variables after the induction of anaesthesia is shown in Figs. 2 and 3. Table 2 shows all haemodynamic data. Both in the sufentanil group and in the remifentanil group MAP, HR, SV, CI, and RPP were significantly decreased 6 min after induction (T_6_) as compared to baseline (T_0_). In contrast, SctO_2_ and SptO_2_ were significantly increased between T_0_ and T_6_. Between-groups comparison (sufentanil vs. remifentanil) of the mean relative changes showed less decrease in: MAP (∆ = − 23 ± 13 vs. -36 ± 13 mmHg; *P* < 0.001), HR (∆ = − 5 ± 7 vs. -10 ± 10 bpm; *P* = 0.025), SV (∆ = − 23 ± 18 vs. -35 ± 19 ml; *P* = 0.017), CI (∆ = − 0.8 (− 1.5 to − 0.5) vs. -1.5 (− 2.0 to − 1.1) l min^− 1^ m^− 2^; *P* = 0.002) and RPP (∆ = − 3077 ± 2010 vs. -5002 ± 2369 mmHg bpm; *P* = 0.001) and a larger increase in SctO_2_ (∆ = 9 ± 5 vs. 6 ± 4%; *P* = 0.040) in the sufentanil group compared to the remifentanil group. The mean relative changes in SptO_2_ (∆ = 8 ± 7 vs. 8 ± 8%; *P* = 0.641) did not differ significantly between groups.

**Table 2:** Haemodynamic data in the sufentanil and remifentanil group after the induction of anaesthesia

		T_0_	T_6_	∆	*P* - value*
MAP (mmHg)	Sufentanil	101 ± 15	78 ± 15	− 23 ± 13	< 0.001
	Remifentanil	100 ± 11	65 ± 11	−36 ± 13	< 0.001
HR (bpm)	Sufentanil	64 ± 9	59 ± 8	−5 ± 7	< 0.001
	Remifentanil	68 ± 14	58 ± 12	− 10 ± 10	0.001
SV (ml)	Sufentanil	101 ± 25	78 ± 21	− 23 ± 18	< 0.001
	Remifentanil	97 ± 21	62 ± 21	− 35 ± 19	< 0.001
CI (l min^− 1^ m^− 2^)	Sufentanil	3.2 (2.7 to 3.9)	2.3 (1.8 to 2.8)	−0.8 (− 1.5 to − 0.5)	< 0.001
	Remifentanil	3.5 (2.7 to 3.9)	1.8 (1.4 to 2.3)	−1.5 (− 2.0 to − 1.1)	< 0.001
RPP (mmHg bpm)	Sufentanil	10,004 ± 2200	6928 ± 1893	−3077 ± 2010	< 0.001
	Remifentanil	10,730 ± 2444	5728 ± 1845	−5002 ± 2369	< 0.001
SctO_2_ (%)	Sufentanil	68 ± 8	77 ± 7	9 ± 5	< 0.001
	Remifentanil	67 ± 7	73 ± 8	6 ± 4	< 0.001
SptO_2_ (%)	Sufentanil	77 ± 10	85 ± 7	8 ± 7	< 0.001
	Remifentanil	72 ± 11	80 ± 9	8 ± 8	< 0.001

Variables are reported as mean ± SD or median (IQR), according to data distribution

∆: value (T_6_) – value (T_0_); *MAP* Mean Arterial Pressure; *HR* Heart Rate; *SV* Stroke Volume; *CO* Cardiac Output; *SctO*_*2*_ Cerebral Tissue Oxygen Saturation; *SptO*_*2*_ Peripheral Tissue Oxygen Saturation

* (T_6_ vs. T_0_), paired *t*-test or Wilcoxon signed rank test

### Haemodynamic effects after the administration of atropine

**Fig. 4:**
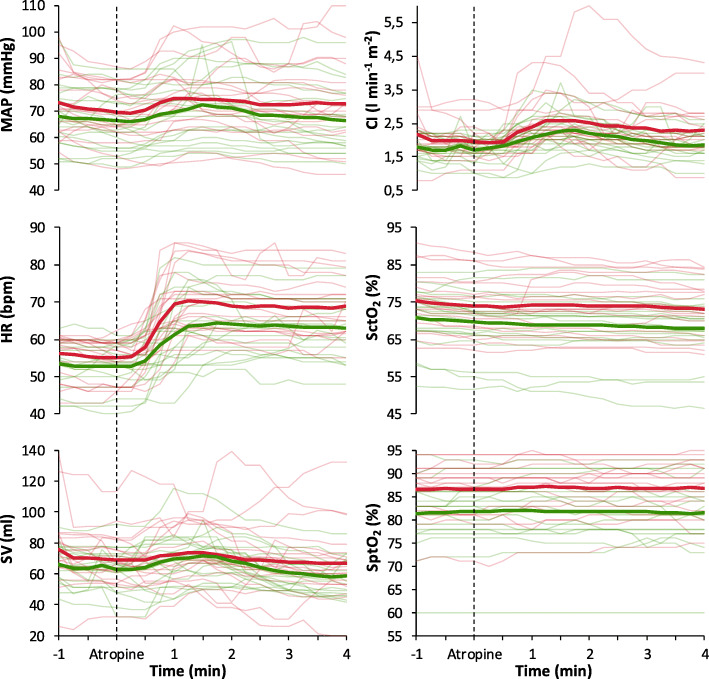
Mean values (thick lines) and individual patient data (thin lines) of the investigated haemodynamic variables: mean arterial pressure (MAP), heart rate (HR), stroke volume (SV), cardiac index (CI), cerebral tissue oxygen saturation (SctO_2_) and peripheral tissue oxygen saturation (SptO_2_). Red lines represent the sufentanil group and green lines the remifentanil group. Graphs are shown from 1 min before until 4 min after the administration of atropine

**Fig. 5:**
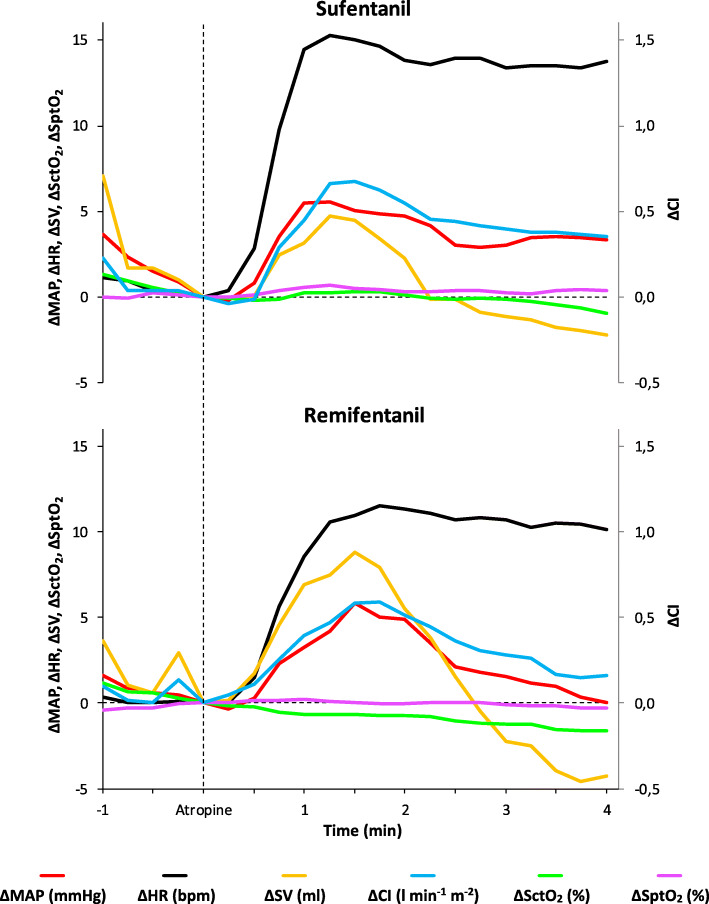
Mean relative changes of the investigated haemodynamic variables during the administration of atropine (from 1 min before until 4 min after). Mean arterial pressure (ΔMAP), heart rate (ΔHR), stroke volume (ΔSV), cardiac index (ΔCI), cerebral tissue oxygen saturation (ΔSctO_2_) and peripheral tissue oxygen saturation (ΔSptO_2_)

Seventy-seven percent of the patients in the sufentanil group and 80 % in the remifentanil group received atropine. The course of the investigated variables after the administration of atropine is shown in Figs. 4 and 5. Table 3 shows all haemodynamic data. In both groups HR, CI, and RPP were significantly increased compared to baseline (T_0_). SctO_2_ significantly decreased compared to baseline (T_0_) in the remifentanil group, but not in the sufentanil group. Between-groups comparison (sufentanil vs. remifentanil) of the mean relative changes showed more increase in: HR (∆ = 13 (9 to 19) vs. 9 (6 to 14) bpm; *P* = 0.016), CI (∆ = 0.4 ± 0.4 vs. 0.2 ± 0.3 l min^− 1^ m^− 2^; *P* = 0.023) and RPP (∆ = 1584 ± 1413 vs. 810 ± 917 mmHg bpm; *P* = 0.027) in the sufentanil group after atropine administration. MAP, SV and tissue oxygenation values were equal in both groups during measurements. Maximum RPP during the measurement period was 12,556 mmHg bpm.

**Table 3:** Haemodynamic data in the sufentanil and remifentanil group after atropine administration

		T_0_	T_4_	∆	*P* - value*
MAP (mmHg)	Sufentanil	69 (63 to 81)	72 (67 to 80)	4 (−2 to 9)	0.101
	Remifentanil	67 (56 to 78)	64 (57 to 77)	1 (−2 to 6)	0.942
HR (bpm)	Sufentanil	57 (49 to 59)	70 (63 to 74)	13 (9 to 19)	< 0.001
	Remifentanil	53 (50 to 58)	64 (56 to 70)	9 (6 to 14)	< 0.001
SV (ml)	Sufentanil	69 ± 17	67 ± 23	−3 ± 10	0.253
	Remifentanil	63 ± 14	59 ± 12	−4 ± 11	0.110
CI (l min^−1^ m^−2^)	Sufentanil	2.0 ± 0.5	2.3 ± 0.8	0.4 ± 0.4	0.001
	Remifentanil	1.7 ± 0.3	1.9 ± 0.4	0.2 ± 0.3	0.032
RPP (mmHg bpm)	Sufentanil	5590 ± 918	7173 ± 1589	1584 ± 1413	< 0.001
	Remifentanil	5204 ± 1053	6014 ± 1590	810 ± 917	0.001
SctO_2_ (%)	Sufentanil	73 (68 to 79)	73 (65 to 78)	−2 (−3 to 1)	0.120
	Remifentanil	72 (66 to 75)	69 (65 to 74)	-2 (−2 to −1)	0.005
SptO_2_ (%)	Sufentanil	88 (81 to 91)	88 (83 to 91)	1 (−1 to 2)	0.582
	Remifentanil	83 (78 to 86)	81 (77 to 86)	0 (−1 to 0)	0.471

Variables are reported as mean ± SD or median (IQR), according to data distribution

∆: value (T_4_) – value (T_0_); *MAP* Mean Arterial Pressure; *HR* Heart Rate; *SV* Stroke Volume; *CO* Cardiac Output; *SctO*_*2*_ Cerebral Tissue Oxygen Saturation; *SptO*_*2*_ Peripheral Tissue Oxygen Saturation

* (T_4_ vs. T_0_), paired *t*-test or Wilcoxon signed rank test

## Discussion

This prospective double-blind randomised controlled trial demonstrates that induction of anaesthesia with propofol and sufentanil results in less haemodynamic suppression than induction of anaesthesia with propofol and a calculated equivalent dosage of remifentanil, combined with more pronounced positive effect on SctO_2_ values in the sufentanil group. Administration of atropine reversed bradycardia and thus maintained haemodynamics and tissue oxygenation with no (clinically) relevant between-groups differences.

After the induction of anaesthesia with comparable doses of opioids, remifentanil did not only cause a more evident decrease in HR, but also in SV. Remifentanil is well known for its direct bradycardic effects, especially compared to other opioids, but not for the negative effect on SV [6, 12]. As a result of a decreased HR, an increase in SV would typically be expected, due to a longer diastolic filling time. This more pronounced decrease in SV in the remifentanil group might be explained by a stronger direct negative inotropic effect of remifentanil, different changes in cardiac preload, or pharmacokinetic differences between both opioids. A larger decrease in HR and SV in the remifentanil group however resulted in a much larger decrease in MAP and CI, and thereby overall more haemodynamic suppression than in the sufentanil group. Furthermore, patients in the remifentanil group demonstrated lower SctO_2_ values. During induction of anaesthesia, a raised inspired oxygen fraction combined with general vasodilation results in an overall increased tissue oxygenation [12]. Despite this, the more pronounced haemodynamic suppression in the remifentanil group led to lower SctO_2_ values than in the sufentanil group. This difference could only be demonstrated in terms of absolute SptO_2_ values, but not in the mean relative values. Also, anaesthesia with more stable haemodynamics, like with sufentanil, will clinically lead to less use of vasoactive medication, e.g. norepinephrine, and thereby less additional haemodynamic suppression and possibly higher tissue oxygenation values [5].

Contrary to our hypothesis, administration of atropine did not result in better attenuation of the haemodynamic suppression caused by remifentanil or higher tissue oxygenation values compared to anaesthesia with sufentanil. Although only demonstrated in absolute values, the sufentanil group showed higher HR and remarkably higher CI values than the remifentanil group. Compared to other opioids, remifentanil evokes a dose dependent prolongation of the cardiac conduction times (by influencing the sinus and atrioventricular node and intra-atrial conduction), inhibition of sinoatrial automaticity and vagally mediated inotropic effects [7, 32]. In accordance with previous observations, we expected more distinct positive effects of atropine on the haemodynamic variables and tissue oxygenation during remifentanil based anaesthesia [12]. However, in our previous study from non-cardiac surgery, none of the patients were on beta-blocking agents, as opposed to the 90% in both of the groups of the current study, which exclusively included patients with ischemic heart disease. Our results show that, not only in patients with extreme bradycardia, atropine significantly improves cardiac index and blood pressure in many cases, which may in clinical practice often obviate the necessity for further interventions such as starting a syringe pump with vasopressive medication. Unfortunately, the methodology of this study did not permit to show any outcome improvement. This was, however not the purpose of the study; the objective was to demonstrate that atropine (especially in remifentanil-based anaesthesia) can induce a significant increase in blood pressure and cardiac output (CO), allegedly by counteracting the parasympathicomimetic effects of the opiates.

Administering atropine in patients undergoing CABG could lead to certain objections, since all of the patients suffer from coronary artery disease. An unwanted increase in HR can result in an increase in the RPP, which is a measure of the internal workload of the heart and a direct indication of the myocardial oxygen demand. However, maximum RPP during the measurement period after the atropine administration was 12,556 mmHg bpm. This value corresponds with a low internal workload and thus indicates a low myocardial oxygen consumption, probably owing to the patients being under anaesthesia [26, 27]. Also, none of the closely monitored patients showed signs of cardiac ischemia. Other contraindications for the use of atropine, like severe aortic valve stenosis, were covered in the exclusion criteria.

There are certain study limitations that need to be addressed. Firstly, equipotent doses of the used opiates are so far not reliably determined in the literature and consequently, we were limited to calculated equipotent doses based on interaction models. Although we consider that these values sufficiently reliably represent clinical reality, different observations may occur depending on opiate doses. Secondly, we used the FloTrac-Vigileo system for the recording of the course of the SV and CI after the induction of anaesthesia and after atropine administration. These variables are calculated via arterial pressure waveform analysis, which leads to a certain inevitable inaccuracy. Previous studies show varying outcomes in agreement and trending ability when FloTrac-Vigileo calculated CO is compared with CO derived from the widely accepted reference method, i.e. thermodilution [19]. Consequently, to minimize measurement bias, we used relative (∆) values instead of absolute values for the comparisons [33, 34].

Also, tissue oxygenation values based on near-infrared spectroscopy and measured during haemodynamic changes, like after induction of anaesthesia, should be interpreted with caution. Reduced skin blood flow and oxygenation, caused by vasoconstrictive medication, hyperventilation and hypoxia, has shown to influence measurement values to some extent [35, 36]. These results however could not be reproduced when norepinephrine was administered to treat postspinal hypotension [37]. Moreover, reduction in SctO_2_ after vasoconstrictive medication has previously been correlated with a decrease in oxygen saturation measured at the level of the jugular venous bulb [38]. As a consequence, we have to assume that the changes in SctO_2_ and SptO_2_ we demonstrated are at least partially caused by changes in skin perfusion. Nevertheless, our data showed a much more evident increase in tissue oxygenation after induction of anaesthesia (6–9% increase), than reported as influence by a change in skin oxygen saturation (2–3% change) [35, 36].

Lastly, for the analysis of the effects of atropine on haemodynamics and tissue oxygenation we used a fixed dose of 1 mg. This could partially account for the (inter-individual) differences in the response of haemodynamic variables to the administration of atropine.

## Conclusions

Induction of anaesthesia with propofol and sufentanil results in improved haemodynamic stability and higher cerebral tissue oxygenation compared to propofol and remifentanil in CABG patients. Administration of atropine might be useful to counteract or prevent the bradycardic action and thus haemodynamic suppression associated with these opioids.

## Data Availability

The datasets used and/or analysed during the current study are available from the corresponding author on reasonable request.

## Abbreviations

bpm: Beats per minute
CABG: Coronary artery bypass grafting
Cet: Effect-site concentration
CI: Cardiac index
CO: Cardiac output
HR: Heart rate
MAP: Mean arterial pressure
RCT: Randomised controlled trial
RPP: Rate pressure product
SctO_2_: Cerebral tissue-oxygenation
SptO_2_: Peripheral tissue-oxygenation
SV: Stroke volume
T_0_: Time = 0 min
T_4_: Time = 4 min
T_6_: Time = 6 min
TCI: Target controlled infusion
